# Supplementary material for: Rapamycin administration causes a decrease in muscle contractile function and systemic glucose intolerance concomitant with reduced skeletal muscle Rictor, the mTORC2 component, expression independent of energy intake in young rats
Source: PLoS One. 2024 Dec 5;19(12):e0312859. doi: 10.1371/journal.pone.0312859 (PMC11620399; doi:10.1371/journal.pone.0312859)
Supplement: S1 Fig — All images were photographed using ChemiDoc XRS+ system (Bio-Rad, Hercules, CA, US). The region shown in the figures were indicated with dashed line. (PDF) [file pone.0312859.s001.pdf]

S1 Fig.Original uncropped images of gel and western blot used for Figs. 2,3,4,5, and 6.

Rapamycin administration causes a decrease in muscle contractile function and systemic glucose intolerance concomitant with reduced skeletal muscle Rictor, the mTORC2 component, expression independent of energy intake in young rats.

Satoru Ato, Chieri Oya, Riki Ogasawara

Rapamycin administration causes a decrease in muscle contractile function and systemic glucose intolerance concomitant with reduced skeletal muscle Rictor, the mTORC2 component, expression independent of energy intake in young rats.

Satoru Ato, Chieri Oya, Riki Ogasawara

Row gel image related to figure 2

Myosin

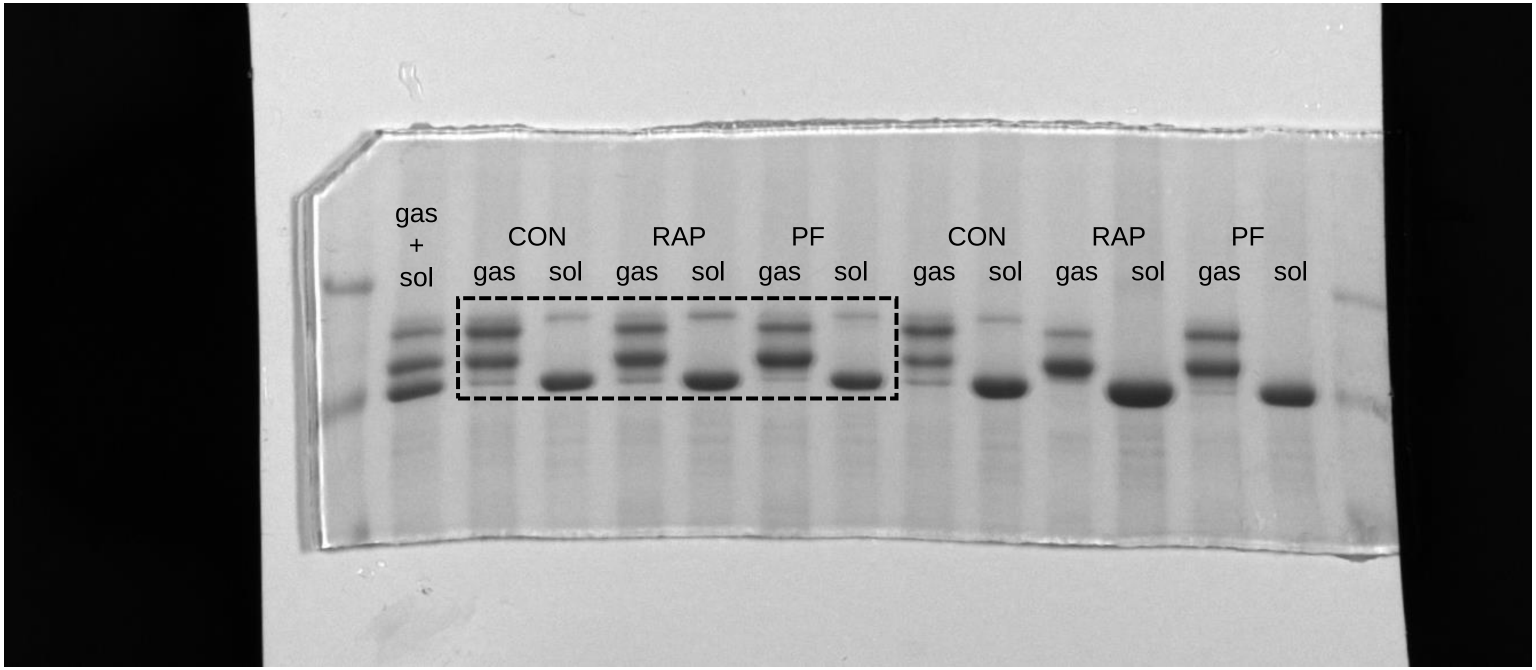

gas
+
sol
CON
RAP
PF
CON
RAP
PF
gas sol gas sol gas sol gas sol gas sol gas sol

Rapamycin administration causes a decrease in muscle contractile function and systemic glucose intolerance concomitant with reduced skeletal muscle Rictor, the mTORC2 component, expression independent of energy intake in young rats.

Satoru Ato, Chieri Oya, Riki Ogasawara

Row blot images related to figure 3

mTOR

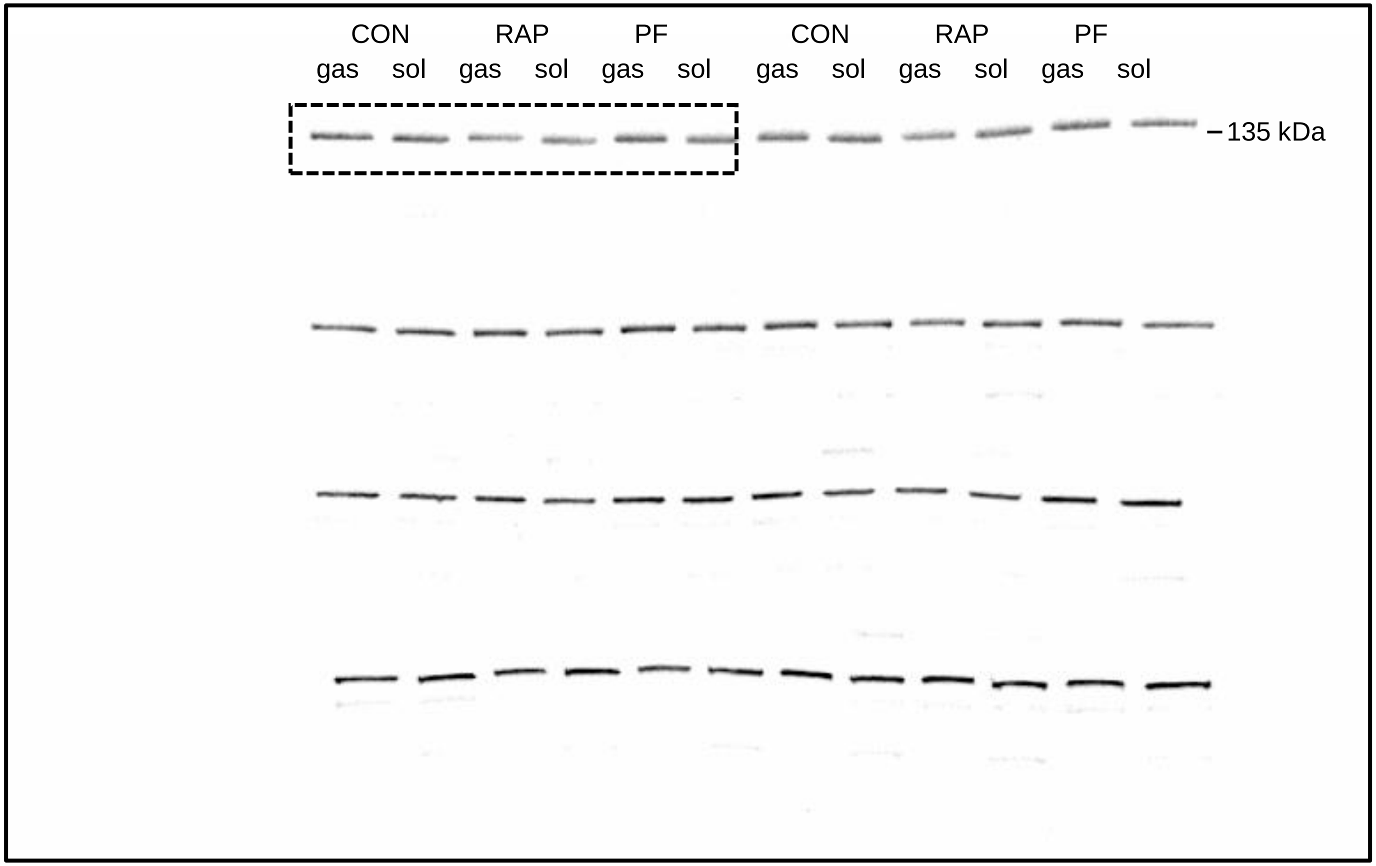

In the western blotting, each gels were cut around region containing target protein and transferred to a PVDF membrane, and antibody reaction was performed to detection.

The protein bands used for figure was annotated with dash lined-square.

Fkbp12

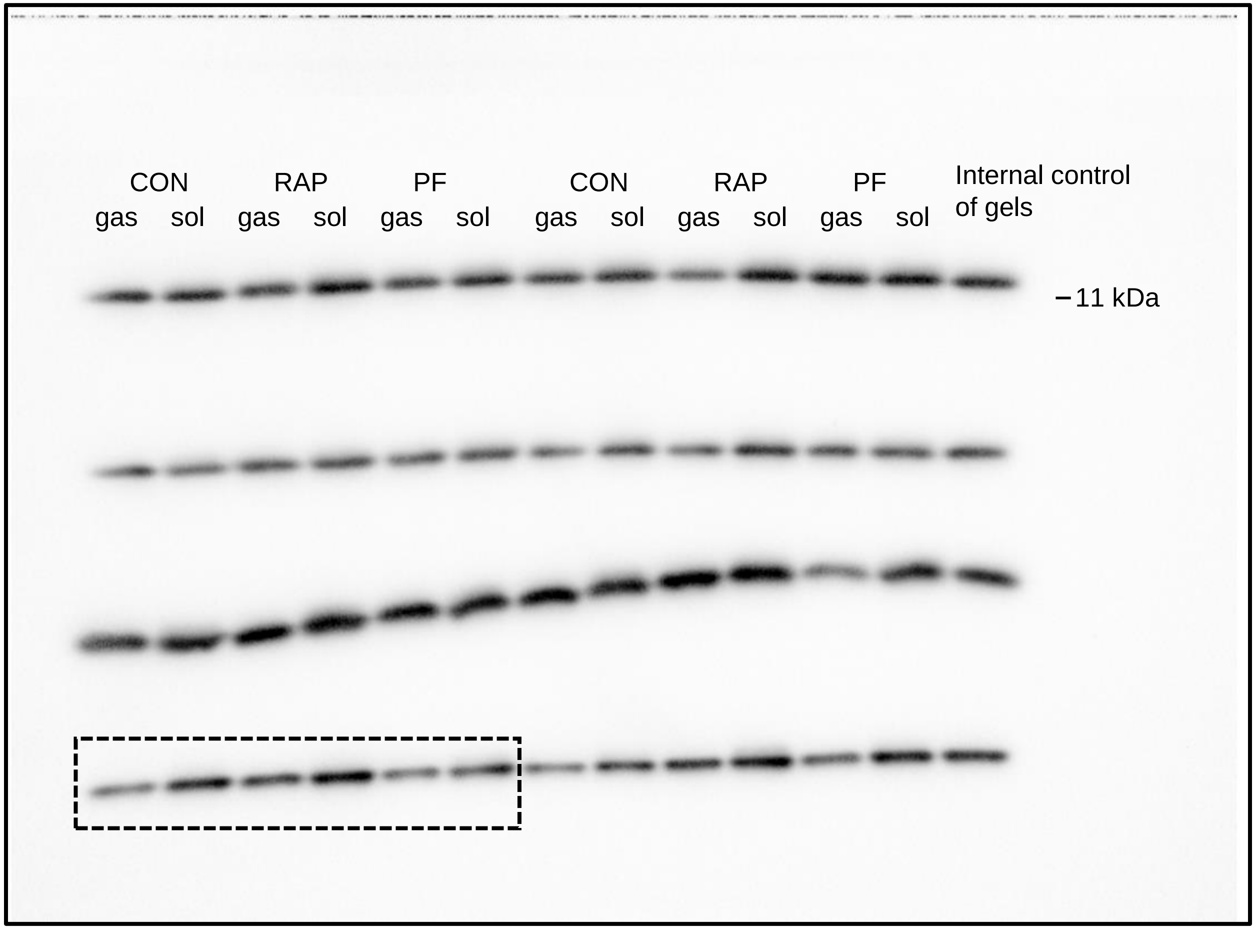

CON
RAP
PF
CON
RAP
PF
Internal control of gels
gas sol gas sol gas sol gas sol gas sol gas sol
11 kDa

Raptor

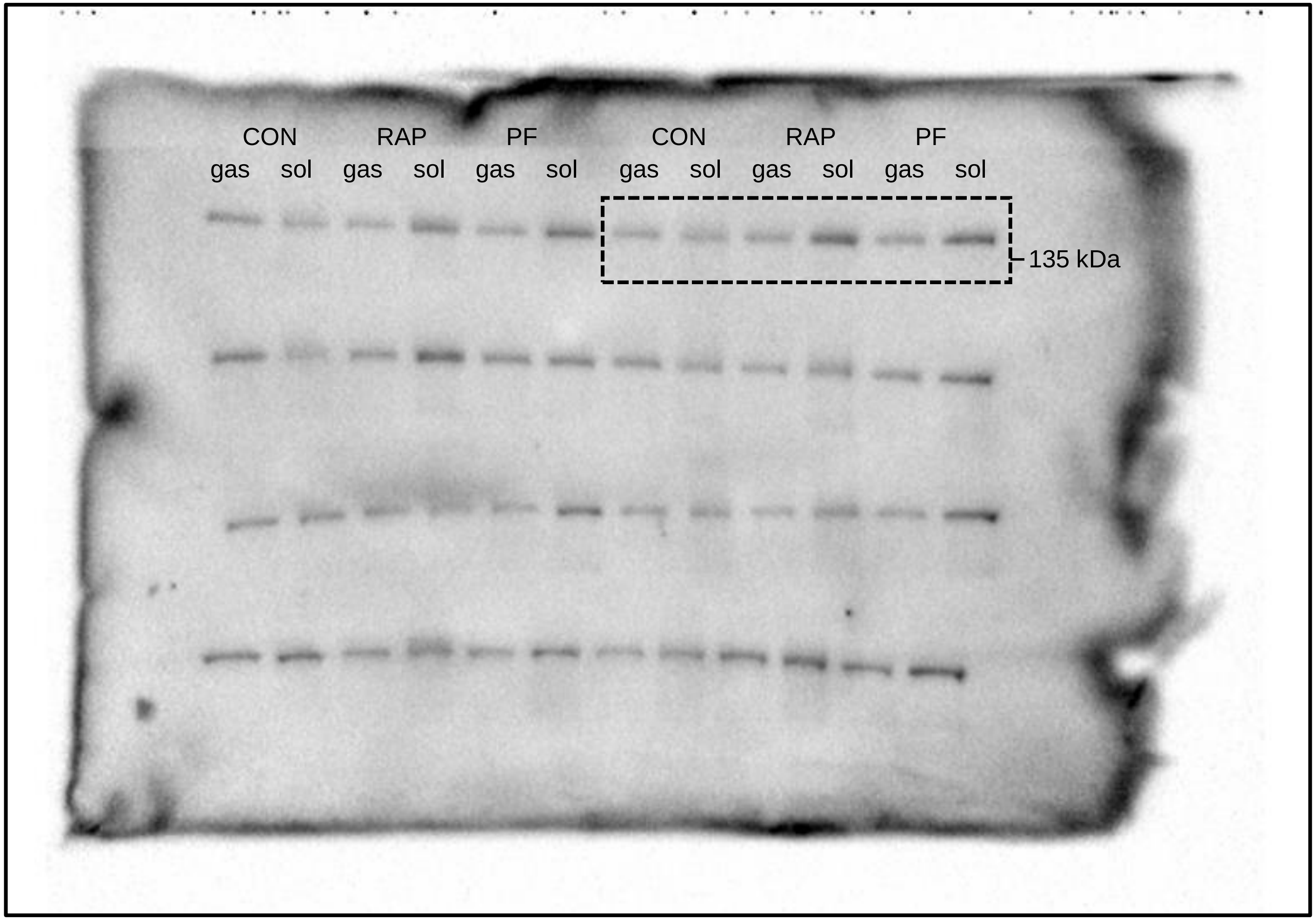
CON
RAP
PF
CON
RAP
PF
gas sol gas sol gas sol gas sol gas sol gas sol
135 kDa

Rictor

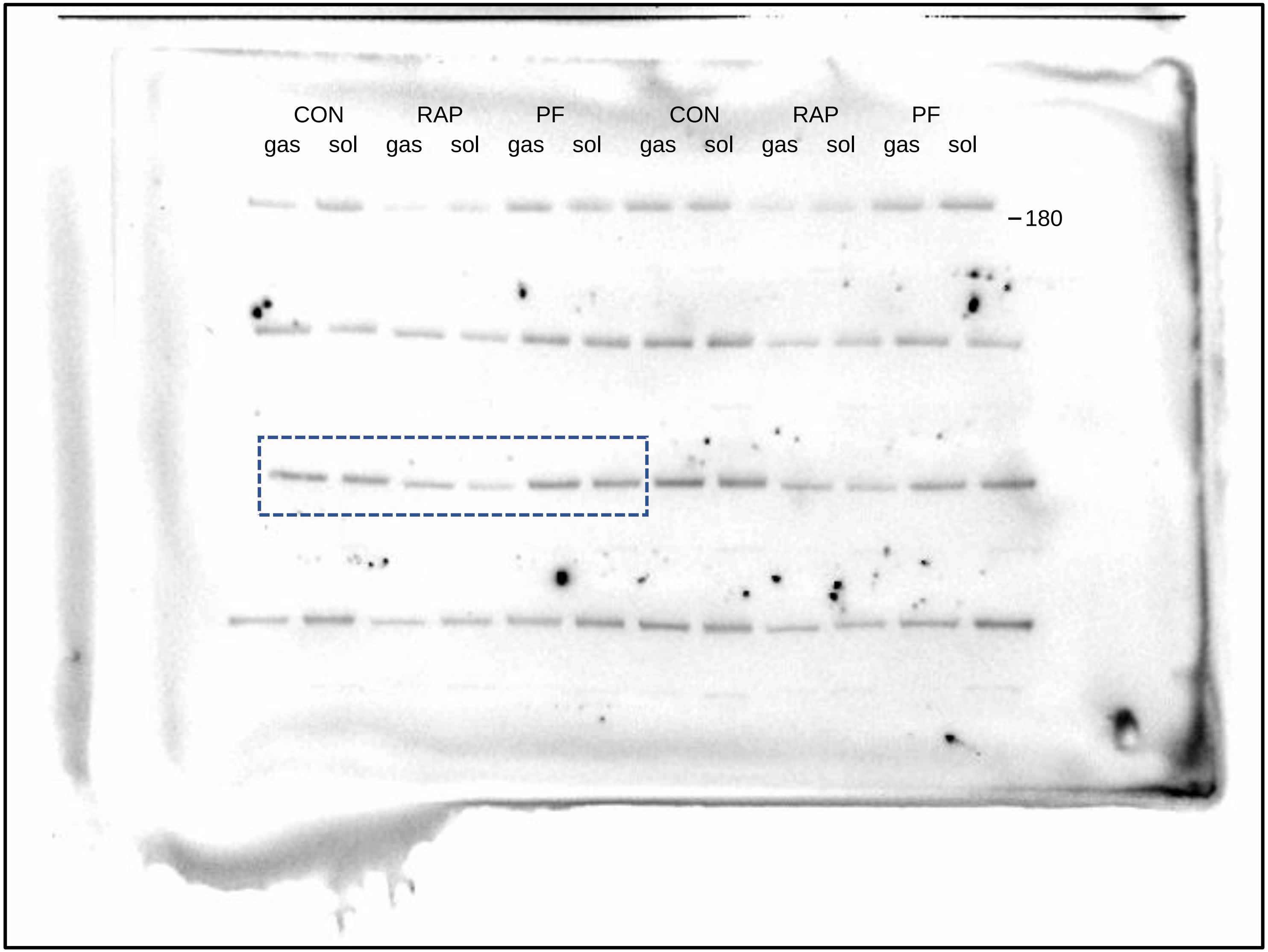
CON
RAP
PF
CON
RAP
PF
gas sol gas sol gas sol gas sol gas sol gas sol
180

p-p70S6K Thr389

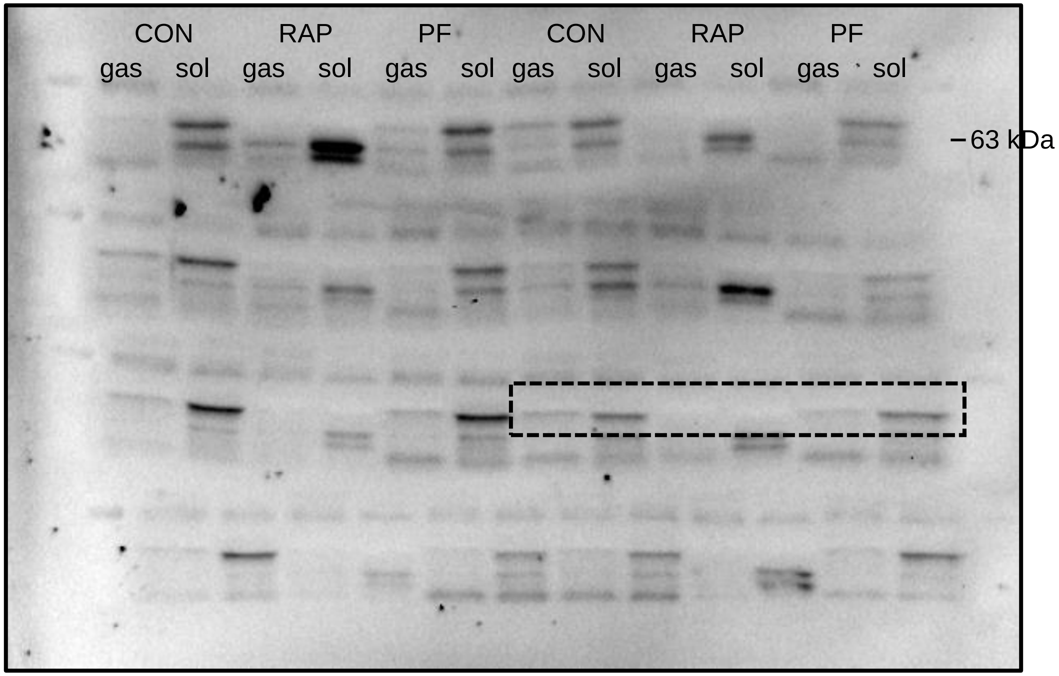

p70S6K

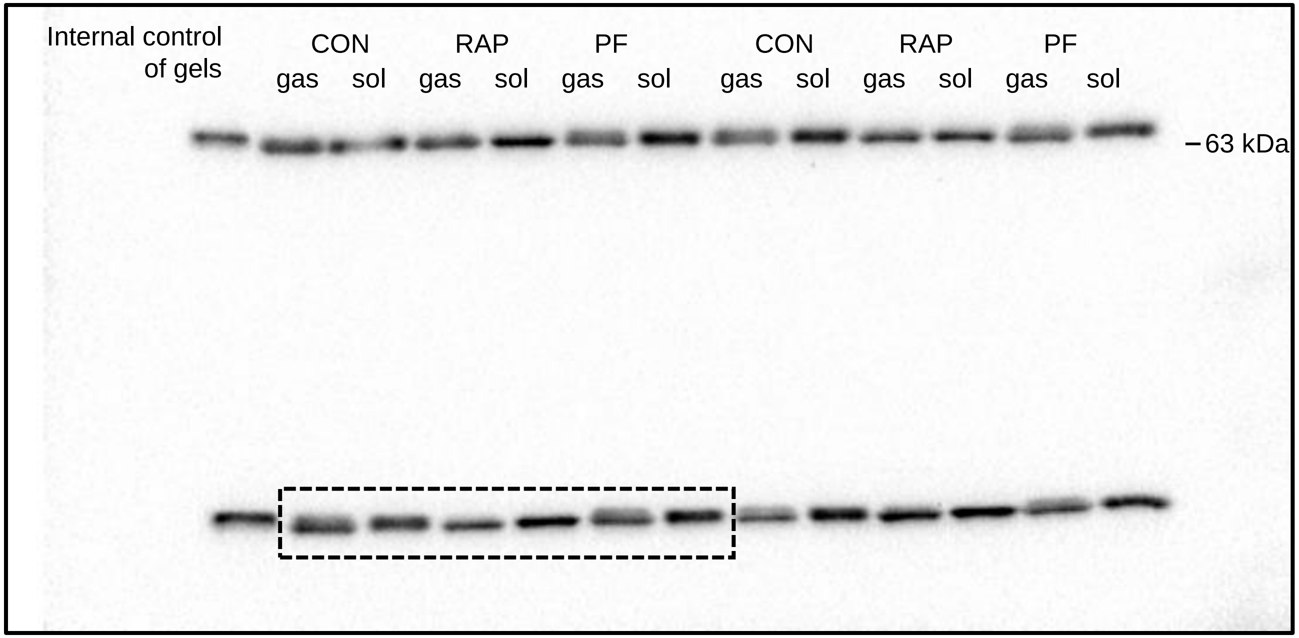

Internal control of gels
CON
RAP
PF
CON
RAP
PF
gas sol gas sol gas sol gas sol gas sol gas sol
63 kDa

p-4E-BP1 Thr37/46

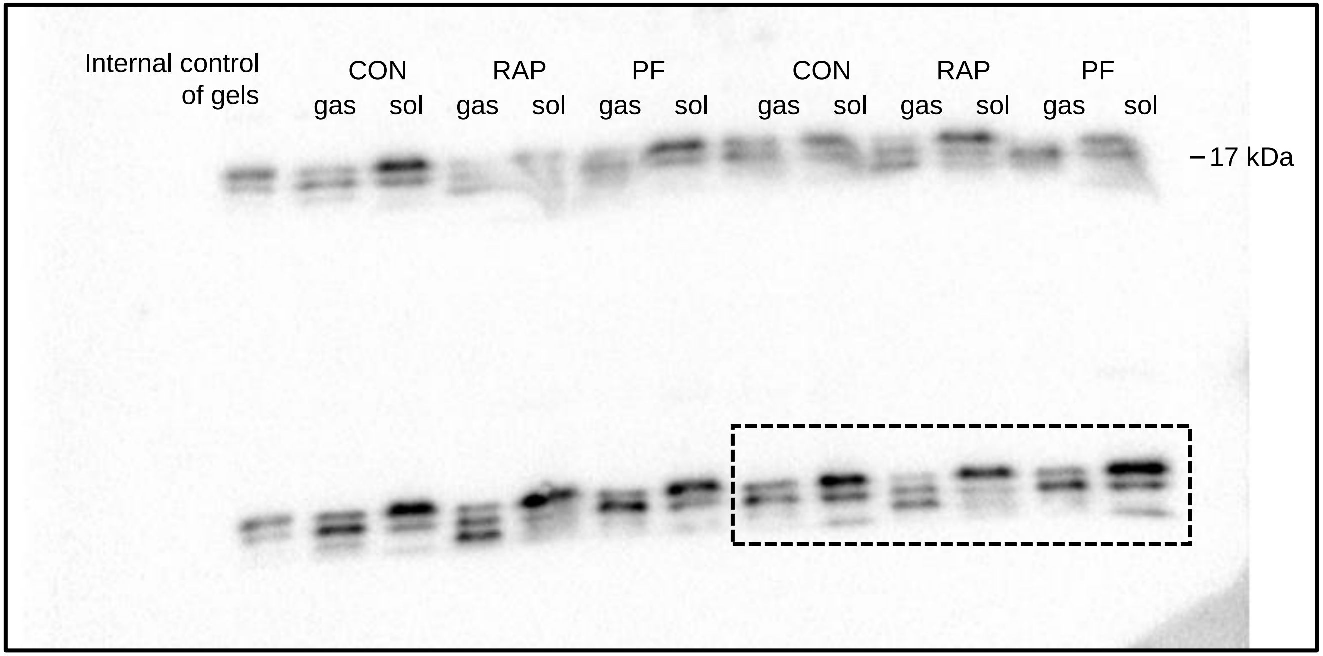

4E-BP1

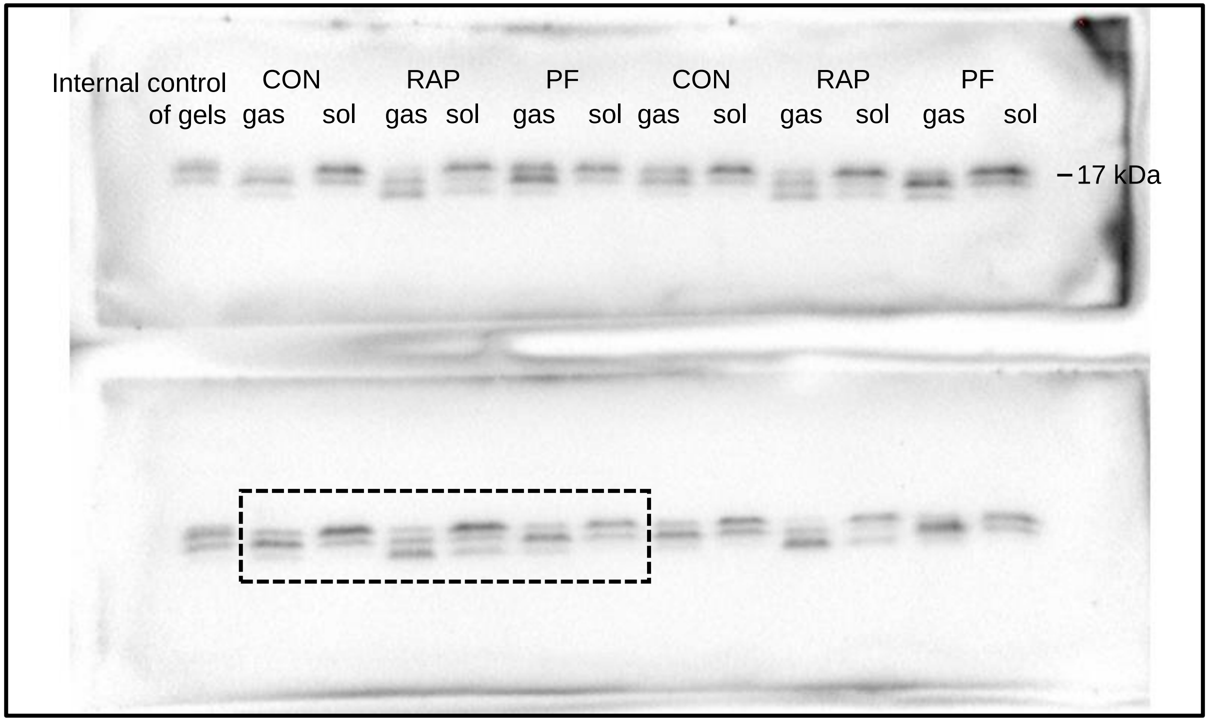
Internal control of gels
CON
RAP
PF
CON
RAP
PF
gas sol gas sol gas sol gas sol gas sol gas sol
17 kDa

p-rpS6 Ser240/244

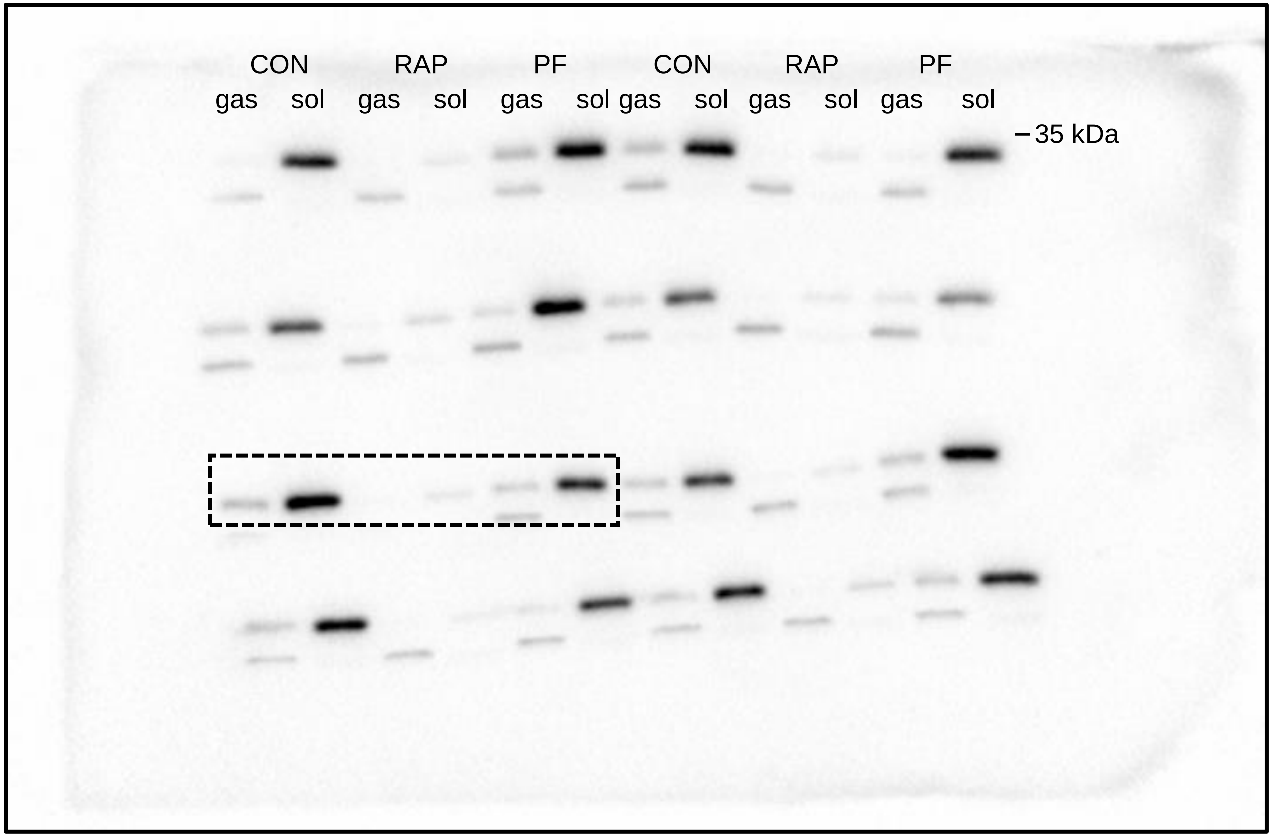

rpS6

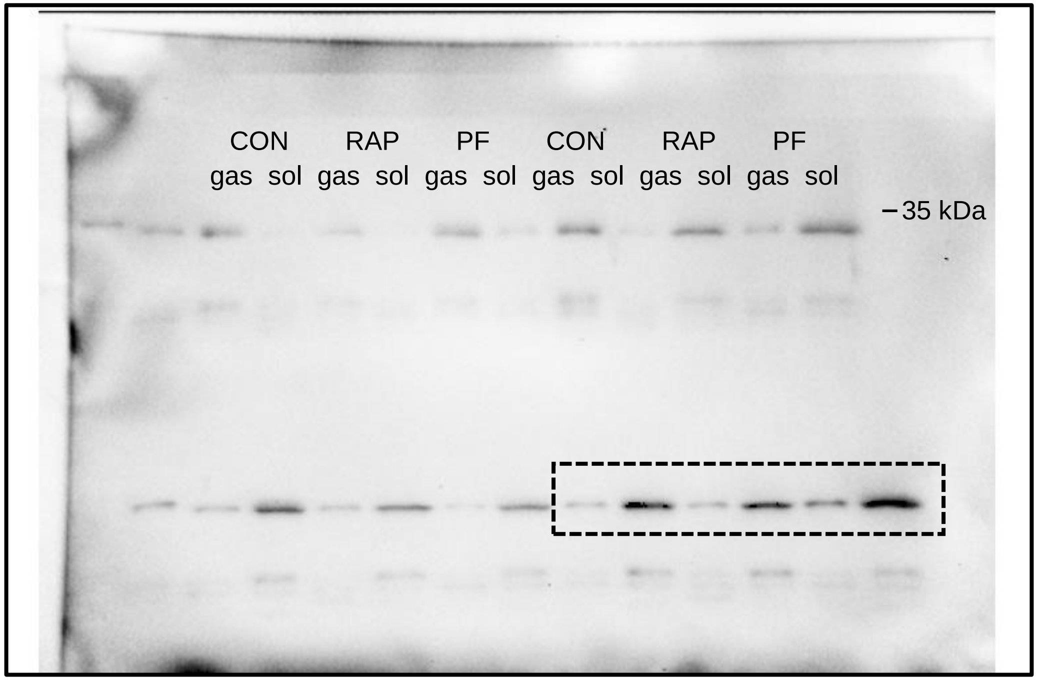
CON RAP PF CON RAP PF
gas sol gas sol gas sol gas sol gas sol gas sol
35 kDa

Rapamycin administration causes a decrease in muscle contractile function and systemic glucose intolerance concomitant with reduced skeletal muscle Rictor, the mTORC2 component, expression independent of energy intake in young rats.

Satoru Ato, Chieri Oya, Riki Ogasawara

Row blots related to figure 4

Puromycin

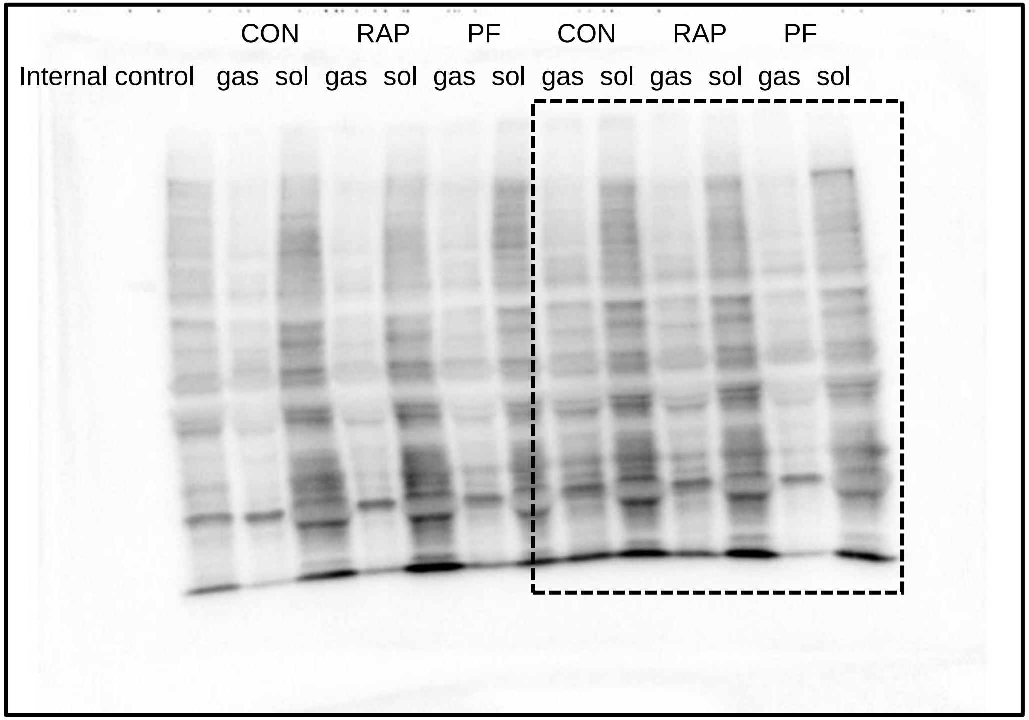
CON RAP PF CON RAP PF
Internal control gas sol gas sol gas sol gas sol gas sol gas sol

Rapamycin administration causes a decrease in muscle contractile function and systemic glucose intolerance concomitant with reduced skeletal muscle Rictor, the mTORC2 component, expression independent of energy intake in young rats.

Satoru Ato, Chieri Oya, Riki Ogasawara

Row blots related to figure 5

LC3

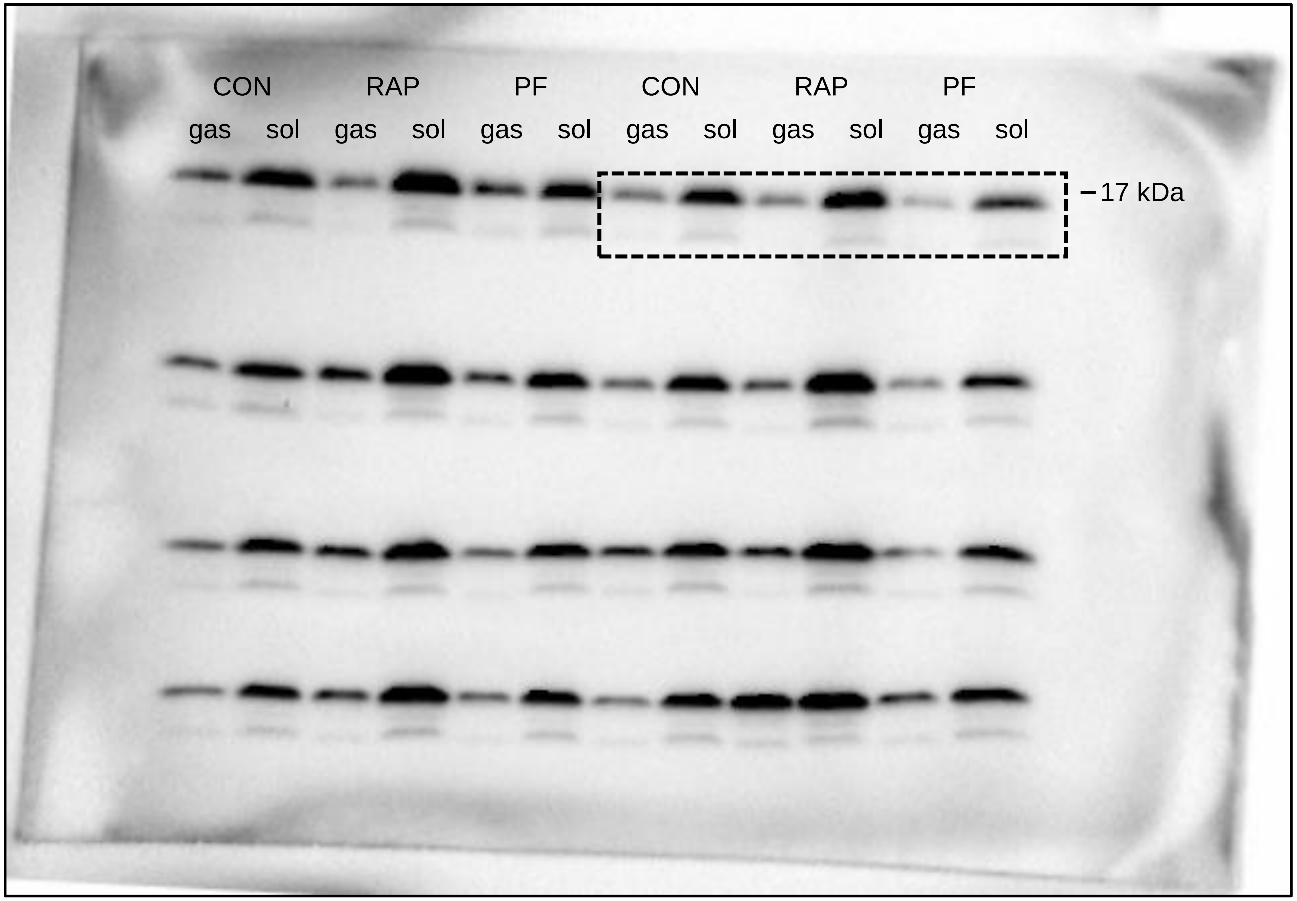
CON
RAP
PF
CON
RAP
PF
gas sol gas sol gas sol gas sol gas sol gas sol
17 kDa

p62

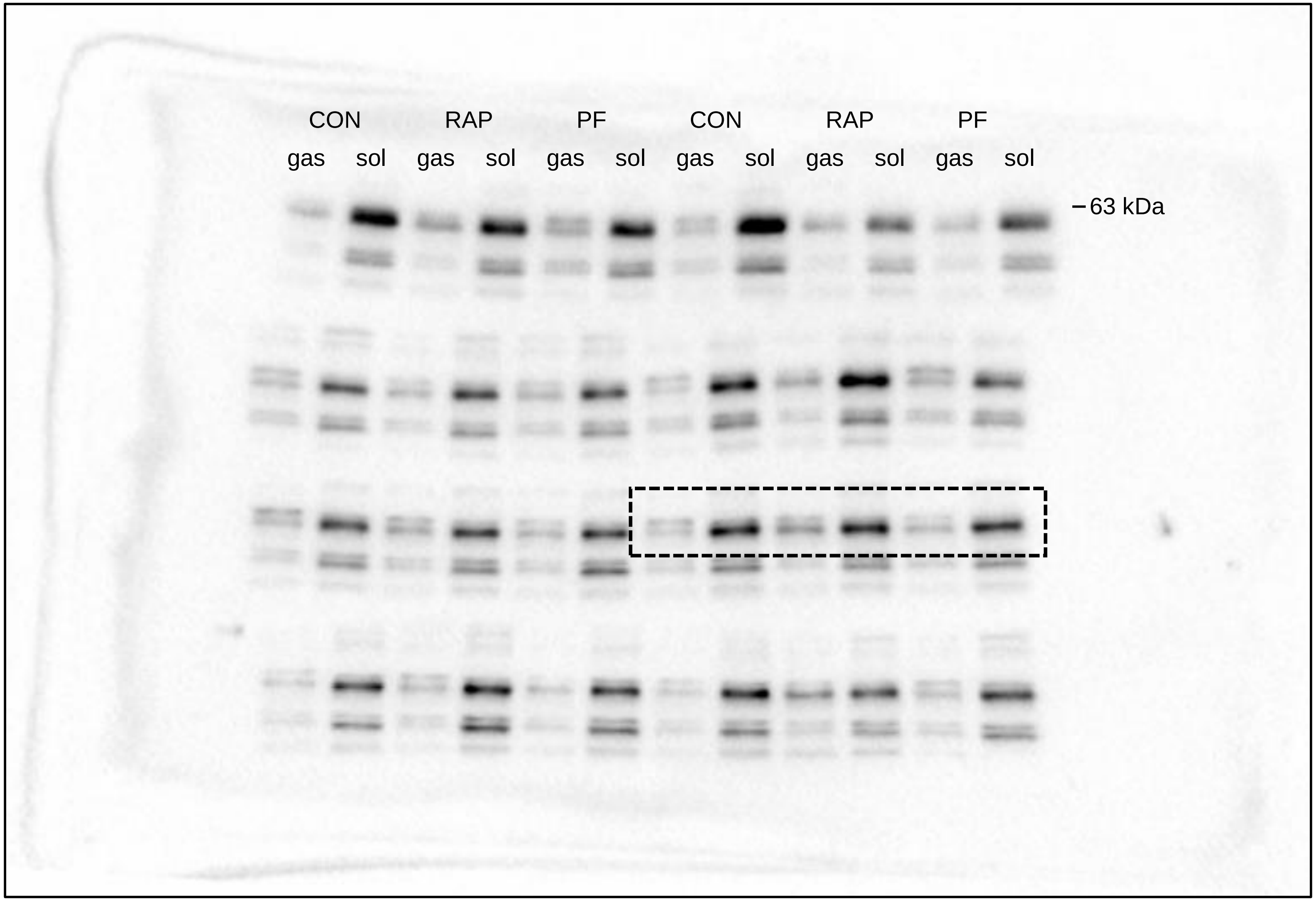
CON
RAP
PF
CON
RAP
PF
gas sol gas sol gas sol gas sol gas sol gas sol
63 kDa

Ubiquitin

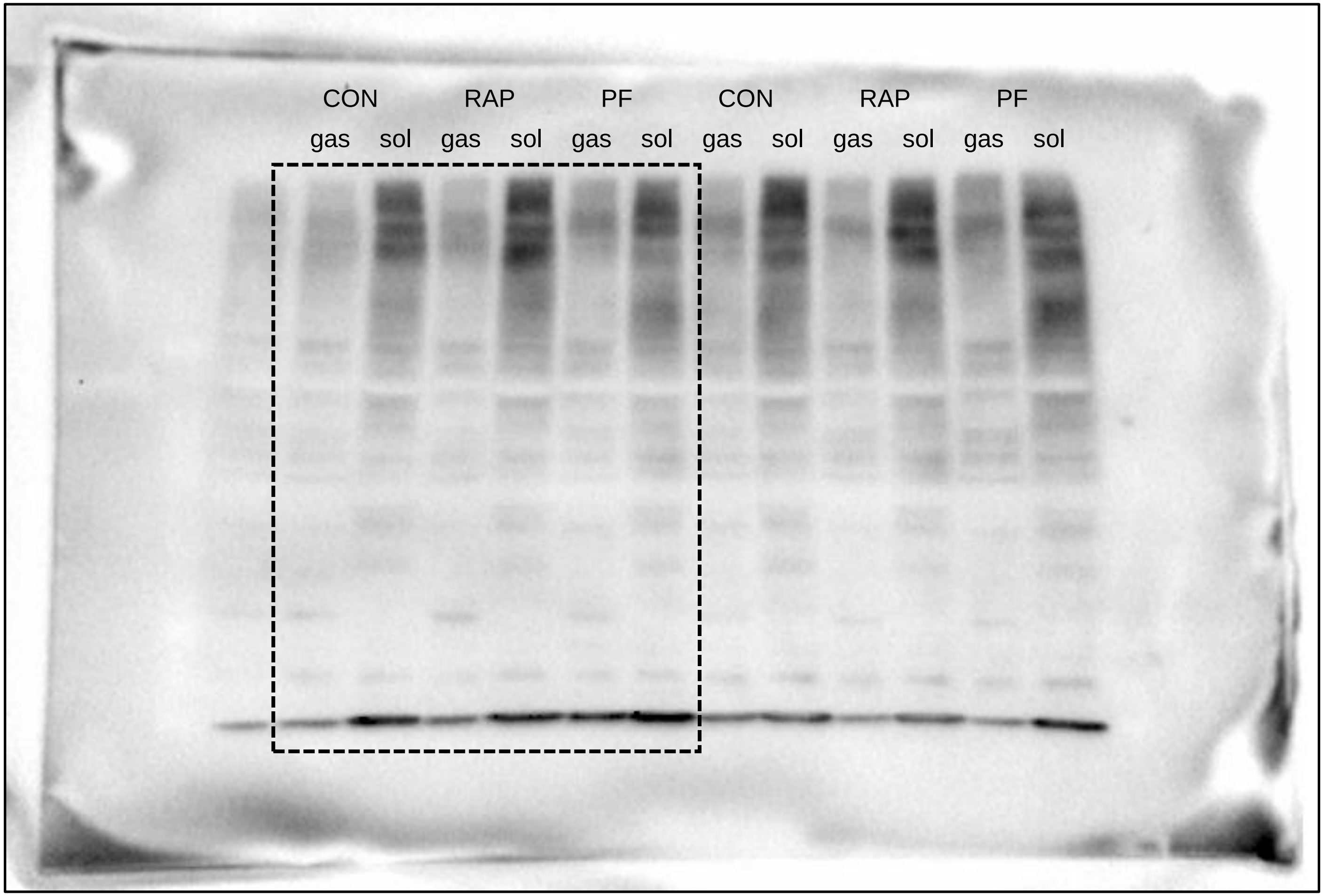
CON RAP PF CON RAP PF
gas sol gas sol gas sol gas sol gas sol gas sol

Rapamycin administration causes a decrease in muscle contractile function and systemic glucose intolerance concomitant with reduced skeletal muscle Rictor, the mTORC2 component, expression independent of energy intake in young rats.

Satoru Ato, Chieri Oya, Riki Ogasawara

Row blots related to figure 6

p-Akt Thr308

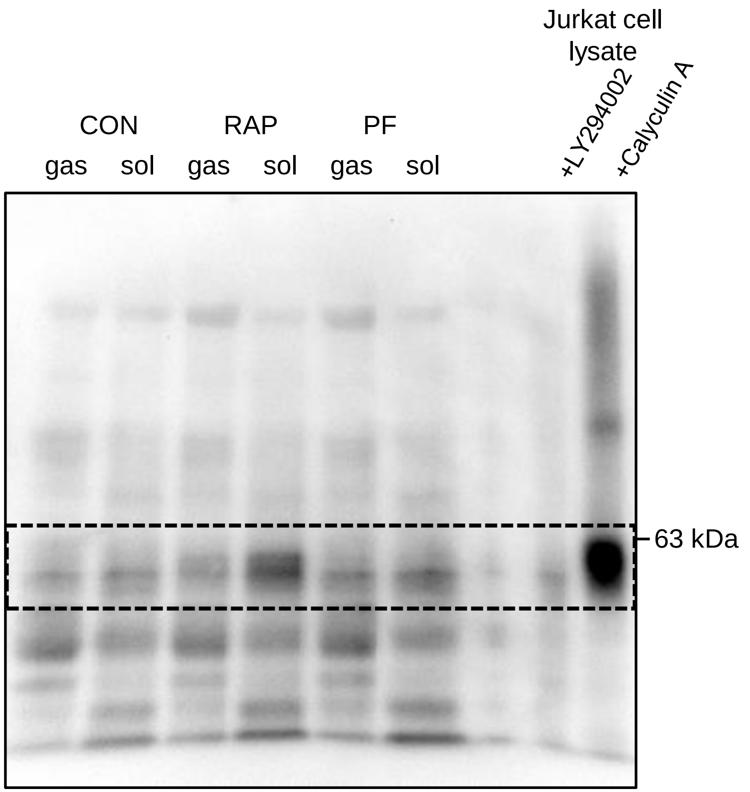
Jurkat cell
lysate
+LY294002
+Calyculin A
CON
RAP
PF
gas
sol
gas
sol
gas
sol
63 kDa

p-Akt Ser473

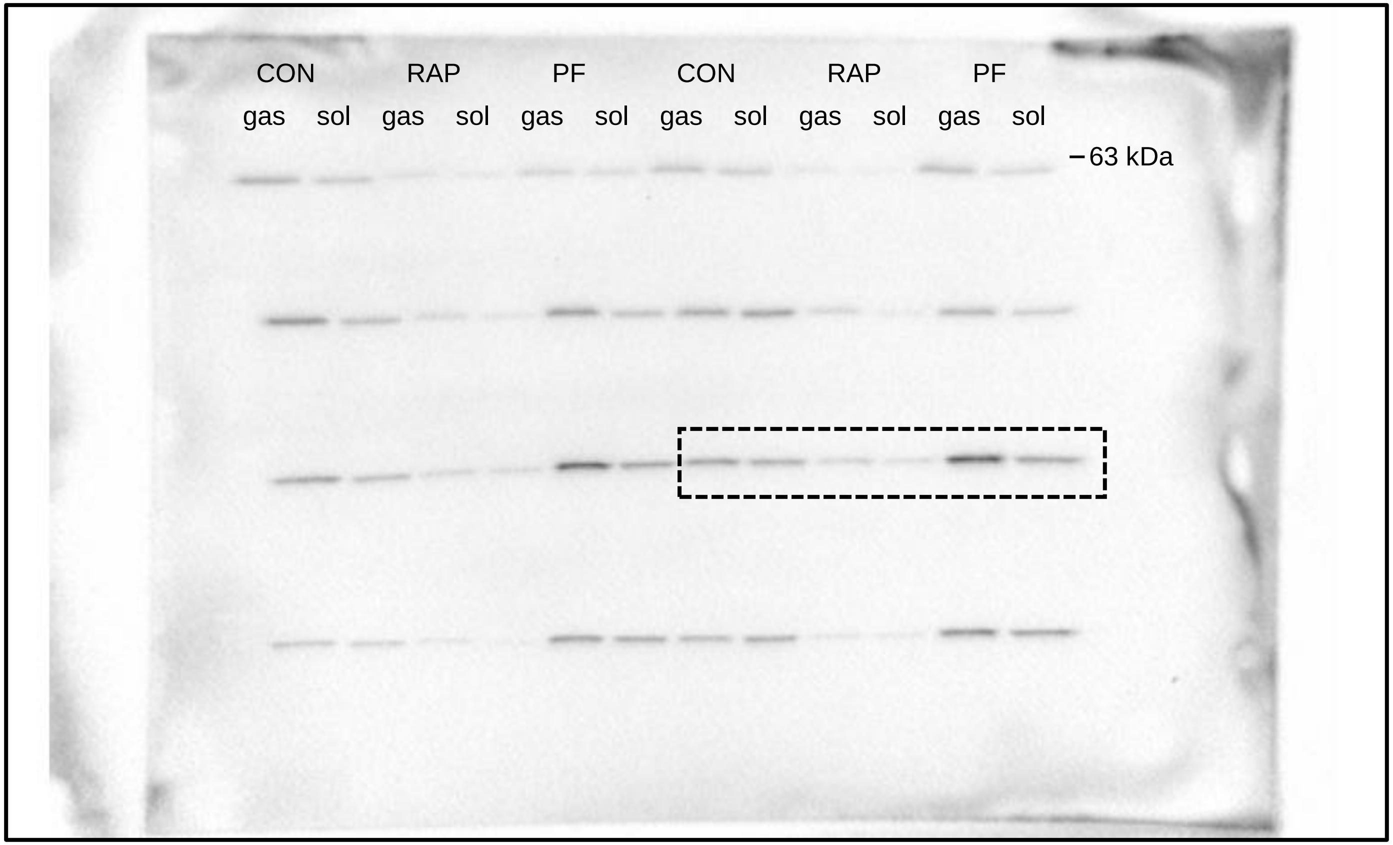

CON RAP PF CON RAP PF
gas sol gas sol gas sol gas sol gas sol gas sol
63 kDa

Akt

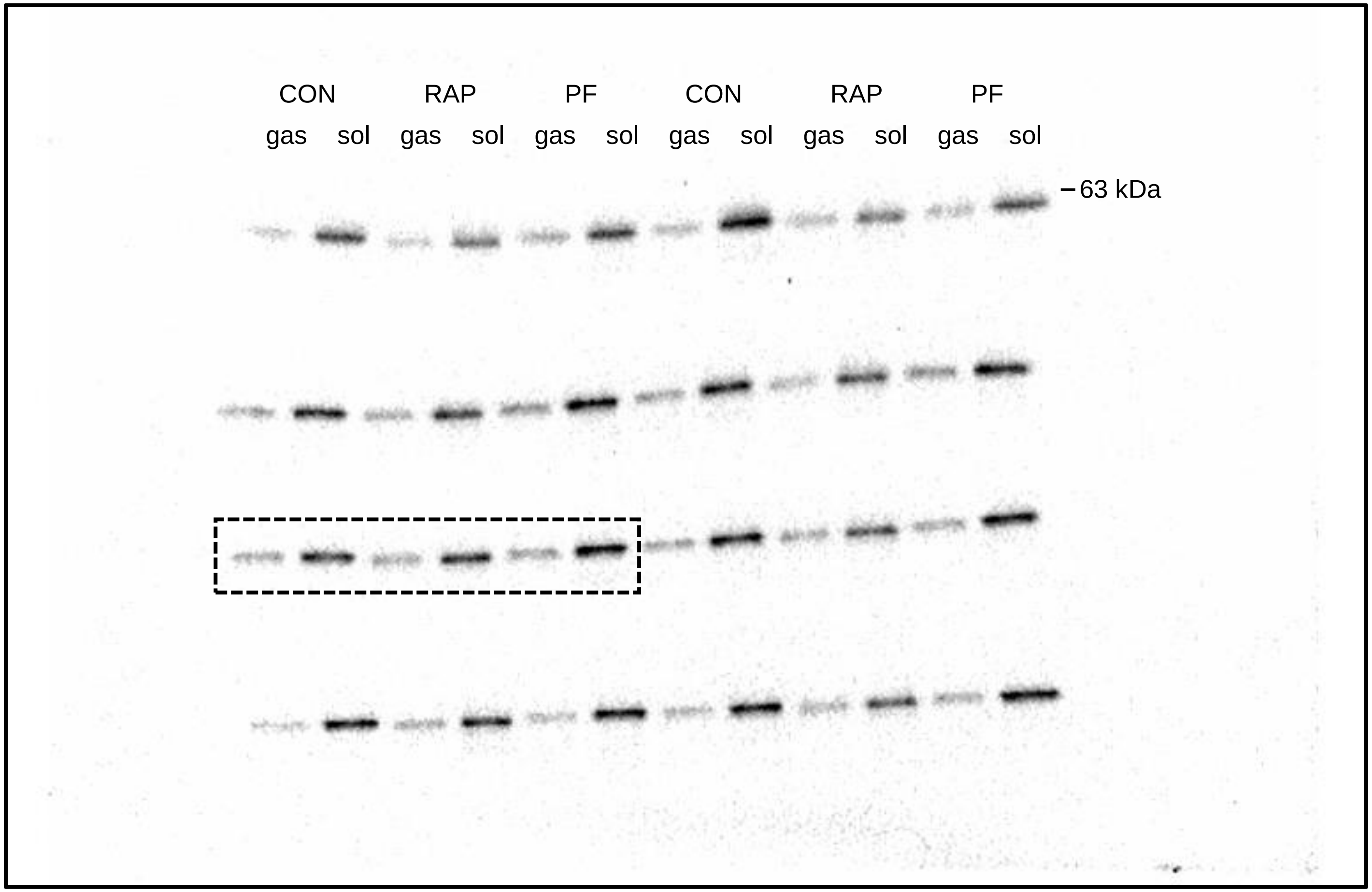

CON RAP PF CON RAP PF
gas sol gas sol gas sol gas sol gas sol gas sol
63 kDa

GLUT4

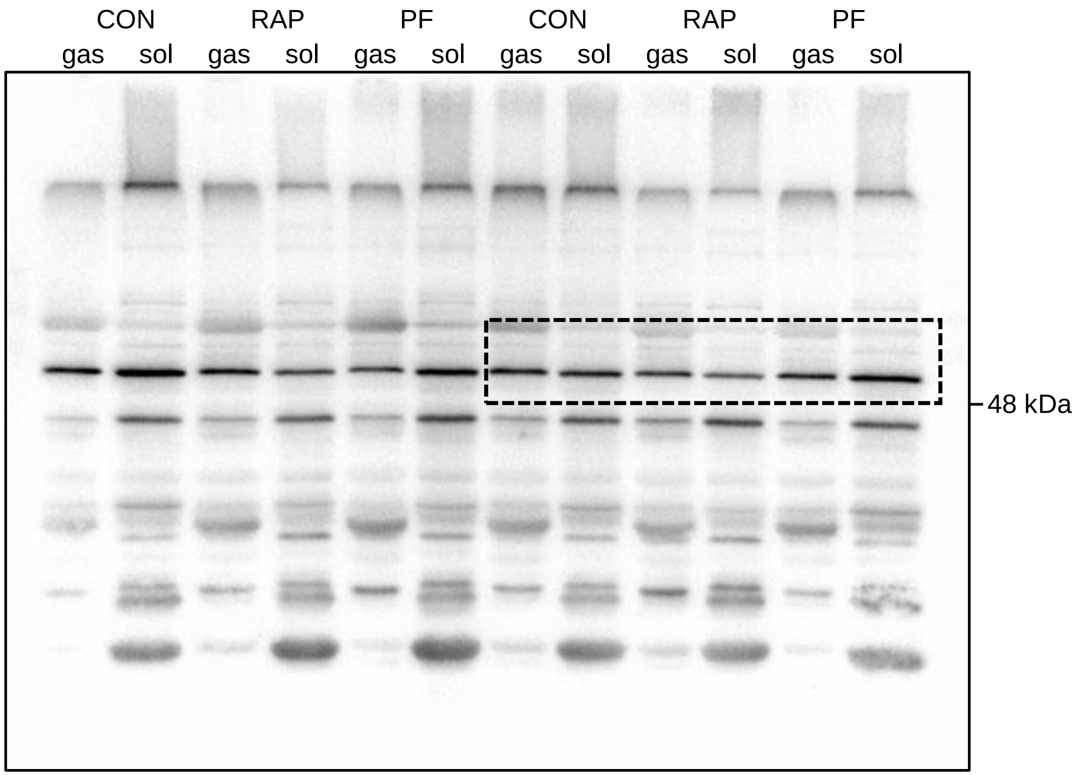

CON
RAP
PF
CON
RAP
PF
gas sol gas sol gas sol gas sol gas sol gas sol
48 kDa

HK2

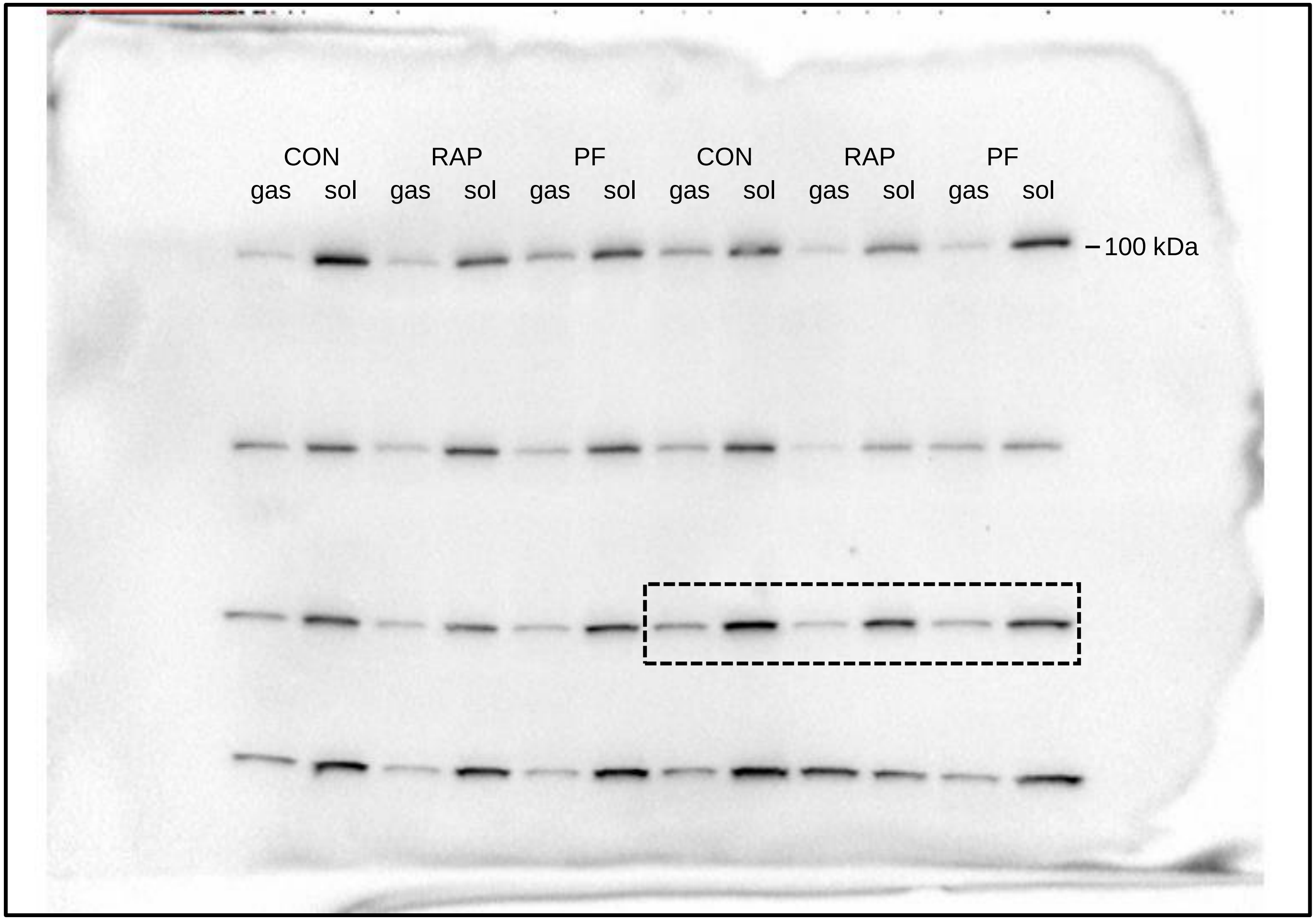
CON RAP PF CON RAP PF
gas sol gas sol gas sol gas sol gas sol gas sol
100 kDa
